# Real-World Outcomes Between Perioperative Chemotherapy (FLOT) and Preoperative Concurrent Chemoradiotherapy (CROSS) in Localized Esophageal and Esophagogastric Junction Adenocarcinoma: A Retrospective Cohort Study

**DOI:** 10.3390/cancers17182962

**Published:** 2025-09-10

**Authors:** Jirapat Wonglhow, Hui-Li Wong, Cuong Duong, John Spillane, David S. Liu, Trevor Leong, Julie Chu, Michael Michael

**Affiliations:** 1 Division of Medical Oncology, Department of Internal Medicine, Faculty of Medicine, Prince of Songkla University, Songkhla 90110, Thailand; 2 Department of Medical Oncology, Peter MacCallum Cancer Centre, Melbourne, VIC 3000, Australia; huili.wong@petermac.org (H.-L.W.); michael.michael@petermac.org (M.M.); 3 Division of Cancer Surgery, Peter MacCallum Cancer Centre, Melbourne, VIC 3000, Australia; cuong.duong@petermac.org (C.D.); john.spillane@petermac.org (J.S.); david.liu@petermac.org (D.S.L.); 4 Division of Cancer Research, Peter MacCallum Cancer Centre, 305 Grattan Street, Melbourne, VIC 3000, Australia; 5 Upper Gastrointestinal Surgery Unit, Division of Surgery, Anaesthesia and Procedural Medicine, Austin Hospital, 145 Studley Road, Heidelberg, VIC 3084, Australia; 6 Victorian Interventional Research and Trials Unit, Department of Surgery, University of Melbourne , Austin Health, 145 Studley Road, Heidelberg, VIC 3084, Australia; 7 Department of Radiation Oncology, Peter MacCallum Cancer Centre, Melbourne, VIC 3000, Australia; trevor.leong@petermac.org (T.L.); julie.chu@petermac.org (J.C.); 8 Sir Peter MacCallum Department of Oncology, University of Melbourne, Melbourne, VIC 3000, Australia

**Keywords:** esophageal cancer, adenocarcinoma, perioperative chemotherapy, preoperative concurrent chemoradiotherapy, FLOT, CROSS, multidisciplinary team

## Abstract

**Background:** The management of localized esophageal and esophagogastric junction (EGJ) adenocarcinomas remains challenging. Although perioperative chemotherapy with the fluorouracil, leucovorin, oxaliplatin, and docetaxel (FLOT) regimen or preoperative concurrent chemoradiotherapy with carboplatin and paclitaxel (CROSS) regimen followed by surgery are standard options, the optimal approach is still debated. This study evaluated real-world outcomes of perioperative FLOT versus preoperative CROSS in such patients. **Methods:** A retrospective cohort study was conducted at a tertiary cancer center in Australia, including patients treated with FLOT or CROSS between 2014 and 2024. Multivariate Cox regression models adjusted for baseline differences, including demographics, tumor stage, differentiation, location, and surgical resection. **Results:** Among 70 patients, 15 received FLOT and 55 received CROSS. Median overall survival (OS) was 30.3 months for FLOT and 37.5 months for CROSS (*p* = 0.75). Median event-free survival (EFS) was not reached in the FLOT group and was 14.8 months in the CROSS group (*p* = 0.49). After multivariate adjustment, differences in OS and EFS were not significant. Compared to FLOT, CROSS was associated with higher treatment completion and response rates. CROSS also led to greater pathological tumor and nodal downstaging, as well as higher rates of complete pathological response. **Conclusions:** Both FLOT and CROSS appear to be effective treatment options for localized esophageal and EGJ adenocarcinomas. CROSS may offer advantages in terms of treatment tolerability and tumor response, and may be particularly suitable for patients with bulky tumors or reduced performance status. Owing to the limited sample size and follow-up, these findings should be interpreted cautiously. Personalized treatment decisions should be guided by multidisciplinary discussions, considering tumor characteristics, patient condition, and access to adjuvant immunotherapy.

## Simple Summary

Esophageal and esophagogastric junction (EGJ) cancers are serious diseases often diagnosed at an advanced stage. Two common treatment approaches are perioperative chemotherapy (before and after surgery) using the FLOT regimen and preoperative chemoradiotherapy (chemotherapy with radiation before surgery) known as the CROSS protocol. This study compared the outcomes of these two treatments in routine clinical practice at a major cancer center in Australia. Both strategies showed similar survival outcomes, but patients who received CROSS had better tumor shrinkage and fewer treatment-related side effects. These findings suggest that CROSS may be a more suitable option for patients who have large tumors or poorer general health, but these findings should be interpreted with caution due to the small sample size and limited follow-up time, especially in the FLOT group. The results highlight that both options remain reasonable, and treatment decisions should be individualized based on patient condition, tumor features, and multidisciplinary input.

## 1. Introduction

Esophageal cancer is the eleventh most common cancer worldwide [1]. According to GLOBOCAN 2024, there were 510,716 new esophageal cancer cases in 2022, with 445,129 deaths, making it the seventh leading cause of cancer-related mortality worldwide. This translates to an estimated mortality rate of 87%, highlighting the poor overall survival (OS) rate associated with this disease [1].

Histologically, esophageal cancer is classified into two primary subtypes: squamous cell carcinoma, which predominantly affects the upper two-thirds of the esophagus, and adenocarcinoma, which typically affects the lower third [2,3]. Although less common, esophagogastric junction (EGJ) cancer is usually an adenocarcinoma subtype and is treated as either esophageal or gastric cancer, depending on its location. Siewert type I and II tumors are generally managed as esophageal adenocarcinomas. The incidence of esophageal adenocarcinoma is increasing in many countries, and obesity, gastroesophageal reflux disease, and Barrett’s esophagus are emerging as key contributors to its increasing burden [1,3].

Most patients are diagnosed at a locally advanced stage, and surgery alone is rarely sufficient [4]. A multimodal approach, which incorporates surgery with chemotherapy and/or radiotherapy, has become the standard of care for resectable diseases [2,5,6,7,8,9,10,11,12]. Preoperative concurrent chemoradiotherapy (CCRT) with carboplatin and paclitaxel, as in the CROSS protocol, has demonstrated improvements in OS and disease-free survival (DFS) compared with surgery alone [13]. Similarly, perioperative chemotherapy has shown superior outcomes compared to surgery alone, with the fluorouracil, leucovorin, oxaliplatin, and docetaxel (FLOT) regimen being the most well-evidenced and standard treatment option for perioperative therapy [14,15,16,17].

Despite these advances, an optimal treatment strategy remains inconclusive [18,19,20,21,22]. While some studies found no significant differences in survival between preoperative chemotherapy and CCRT, preoperative CCRT appears to improve pathological complete response (pCR) and R0 resection rates without significantly affecting survival [23,24,25]. In contrast, a recent study comparing FLOT-based perioperative chemotherapy with preoperative CCRT suggested that perioperative treatment may provide superior OS, DFS, and pCR [26]. However, these conflicting results may reflect differences in patient selection as well as treatment delivery and highlight the complexity of treatment decision making. Notably, previous studies did not incorporate the current standard of adjuvant immunotherapy for patients with pathological residual disease following preoperative CCRT [27].

Given the variety of treatment options available, we aimed to evaluate real-world outcomes of perioperative chemotherapy (FLOT) and preoperative CCRT (CROSS) in patients with localized esophageal and EGJ adenocarcinomas to better inform clinical decision making in contemporary practice.

## 2. Materials and Methods

We retrospectively reviewed the medical records of newly diagnosed patients with esophageal adenocarcinoma at the Peter MacCallum Cancer Centre, Australia, between January 2014 and December 2024. Eligible patients met the following inclusion criteria: (1) histologically confirmed adenocarcinoma of the esophagus or EGJ classified as Siewert type I or II; (2) clinical stage cT2N0 or cT1–4N+ without evidence of distant metastases (M0), based on computed tomography (CT), fluorodeoxyglucose (FDG) positron emission tomography (PET), and diagnostic laparoscopy; (3) patients planned for curative surgical resection following neoadjuvant treatment with either perioperative chemotherapy using the FLOT regimen or preoperative CCRT with weekly carboplatin and paclitaxel and 41.4 Gy of radiation in accordance with the CROSS protocol; and (4) aged ≥18 years. Patients were excluded if they had a history of prior chemotherapy or radiotherapy, if they were concurrently diagnosed with another active malignancy, or if they were planned for definitive chemoradiotherapy without surgery. The flow diagram of patient selection is presented in Figure S1.

Patient data were retrieved from electronic medical records using the EPIC system at the Peter MacCallum Cancer Centre. Baseline information included demographic and clinical variables such as age, sex, Eastern Cooperative Oncology Group (ECOG) performance status (PS), body mass index (BMI), comorbidities, and initial laboratory findings. Tumor-specific data, including TNM staging (as per the 8th edition of the American Joint Committee on Cancer), histological differentiation, and relevant tumor biomarkers, were also recorded. Treatment-related details, such as chemotherapy protocols, surgical interventions, radiotherapy, and any subsequent therapies, were compiled.

The study was conducted in accordance with the Declaration of Helsinki and approved by the Human Research Ethics Committee of the Peter MacCallum Cancer Centre (HREC QA/117060/PMCC). Given the retrospective design of the study, the requirement for written informed consent was waived. All personally identifiable information was removed to ensure confidentiality.

The FLOT chemotherapy regimen was administered as follows: on day 1 of each cycle, the patients received intravenous infusions of docetaxel (50 mg/m^2^), oxaliplatin (85 mg/m^2^), and leucovorin (200 mg/m^2^), followed by a 24 h continuous intravenous infusion of 5-fluorouracil (5-FU) at a dose of 2600 mg/m^2^. The treatment cycles were repeated every 2 weeks. Patients received four cycles prior to surgery (preoperative phase), with an additional four cycles administered postoperatively (adjuvant phase). Granulocyte colony-stimulating factor (G-CSF) was routinely used as primary prophylaxis. In the CROSS chemoradiotherapy protocol, patients were administered paclitaxel (50 mg/m^2^) and carboplatin (AUC 2) as intravenous infusions on days 1, 8, 15, 22, and 29. Concurrent radiotherapy was delivered at a total dose of 41.4 Gy in 23 fractions (1.8 Gy each) administered 5 days per week over 5 weeks. The chemotherapy dose was adjusted by primary oncologists based on the patient’s ECOG PS and baseline laboratory values and institutional guidelines. The treatment was discontinued prematurely in cases of unacceptable toxicity, disease progression, patient withdrawal, or death.

### 2.1. Objectives and Endpoints

The primary objective of this study was to compare OS between perioperative FLOT and preoperative CROSS in patients with localized esophageal and EGJ adenocarcinomas. Secondary objectives included comparison of EFS, objective response rate (ORR), pCR rate, and treatment-related toxicities between the two treatment groups. OS was defined as the time from the initiation of chemotherapy to death from any cause. EFS was measured from the start of chemotherapy to the first occurrence of radiologically confirmed tumor recurrence or death, whichever occurred first. Response assessment was performed using contrast-enhanced CT of the chest and abdomen and FDG PET. Response rate was evaluated using the Positron Emission Tomography Response Criteria in Solid Tumors (PERCIST) criteria [28]. The imaging results were reviewed and confirmed during multidisciplinary team meetings involving radiologists and nuclear medicine specialists. Both CT and PET imaging were performed after completion of preoperative chemotherapy or CCRT and prior to surgical intervention.

### 2.2. Statistical Analysis

Analysis was based on an intention-to-treat basis. For baseline characteristics, continuous variables are summarized using medians with interquartile ranges (IQRs) or means with standard deviations (SDs), depending on the data distribution. Categorical variables are reported as counts and corresponding percentages. Survival analyses were conducted using the Kaplan–Meier method, and differences between groups were assessed using the log-rank test. To account for potential confounders, multivariate analysis was performed using the Cox proportional hazards model. All statistical analyses were conducted using the R software (version 4.4.3; R Foundation for Statistical Computing, Vienna, Austria). Statistical significance was set at *p* <0.05.

## 3. Results

### 3.1. Baseline Characteristics

Between January 2014 and December 2024, 70 patients with localized esophageal and EGJ adenocarcinomas were enrolled. Of these, 15 (21.4%) underwent perioperative FLOT and 55 (78.6%) underwent preoperative CROSS. The baseline patient characteristics are summarized in Table 1. There was a trend toward a better PS (ECOG PS 0) among patients receiving FLOT than among those treated with CROSS. Differences in clinical T (cT) stage, primary tumor location, and use of preoperative feeding tube support between groups were observed. In terms of tumor location, 93% of the tumors in the FLOT group were in the EGJ (Siewert type II), whereas for the CROSS group, they were more commonly in the distal esophagus (48.1%). Tumor differentiation was similar in both groups. Preoperative feeding tube insertion was more frequent in the CROSS group than in the FLOT group.

### 3.2. Treatment Information

Among the patients who received the FLOT regimen, 93.3% completed all four planned cycles of preoperative chemotherapy, whereas 46.7% completed both the preoperative and postoperative components. In the CROSS group, 92.7% of the patients completed the planned five cycles of chemotherapy during preoperative chemoradiotherapy. Treatment discontinuation was attributable to several factors (Table 2). In the FLOT group, one patient died following the completion of neoadjuvant chemotherapy due to aortic root and brain abscesses. Another patient died postoperatively due to anastomotic leakage complicated by hospital-acquired pneumonia and sepsis.

Radical surgical resection was performed in 86.7% and 67.3% of the patients in the FLOT and CROSS groups, respectively. The most common reason for not undergoing resection in the CROSS group was deterioration in the ECOG PS, which was reported in 14.5% of the patients. Furthermore, five patients (9.1%) in the CROSS group developed metastatic disease before surgery.

Adjuvant nivolumab was administered to 10.9% of the patients in the overall CROSS group. Among patients who underwent surgical resection and had residual pathological disease (non-pCR), 19.4% (6 of 31) received adjuvant nivolumab, reflecting the regulatory environment at the time.

### 3.3. Effectiveness

#### 3.3.1. OS

**Figure 1:**
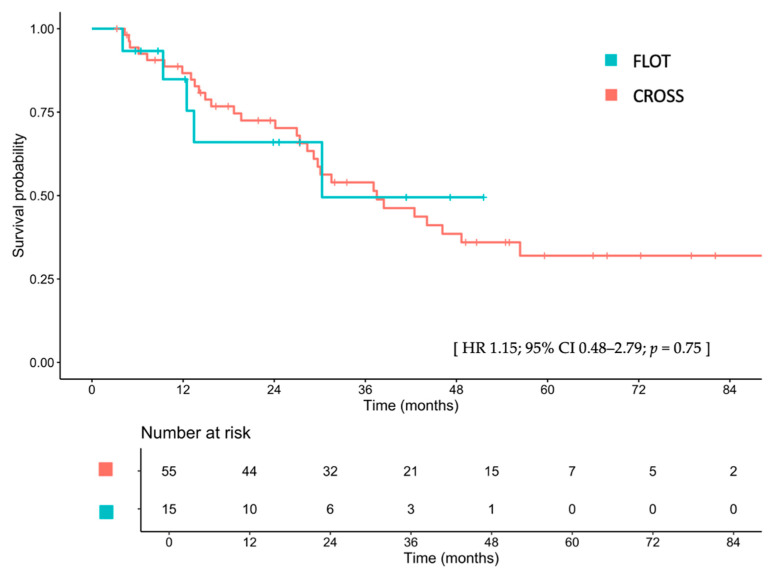
Overall survival between patients treated with FLOT and those treated with CROSS. FLOT, fluorouracil, leucovorin, oxaliplatin, and docetaxel; CROSS, chemoradiotherapy for esophageal cancer followed by surgery study; CI, confidence interval; HR, hazard ratio.

The median follow-up duration for the entire cohort was 27.2 months (IQR 12.6–45.6). Median follow-up times for the FLOT and CROSS groups were 13.4 months (IQR 7.9–28.8) and 28.4 months (IQR 14.2–48.9), respectively. Median OS was 30.3 months (95% CI, 12.5–not reached [NA]) in the FLOT group and 37.5 months (95% CI, 29.2–NA) in the CROSS group, with a hazard ratio (HR) of 1.15 (95% CI, 0.48–2.79; *p* = 0.75; Figure 1). After adjustments for sex, age, ECOG PS, clinical T stage, clinical N stage, tumor differentiation, tumor location, and receipt of surgical resection, the adjusted HR for OS was 2.90 (95% CI, 0.66–12.8; *p* = 0.16). Detailed univariate and multivariable Cox proportional hazard analyses are provided in Table S1. The 1-, 2-, and 3-year OS rates for the FLOT group were 78.8%, 61.3%, and 46.0%, respectively, compared to 86.7%, 72.5%, and 54.0% in the CROSS group.

Among patients treated with CROSS who had residual disease (non-pCR), those who received adjuvant nivolumab had a median OS that was not reached versus 44.1 months for those who did not receive nivolumab (HR 0.61; 95% CI, 0.08–4.75; *p* = 0.633; Figure S2). For all patients undergoing resection in the CROSS group, median OS was 44.1 months compared to 19.6 months in those who did not undergo resection (HR 0.58; 95% CI, 0.28–1.24; *p* = 0.16; Figure S3).

#### 3.3.2. EFS

**Figure 2:**
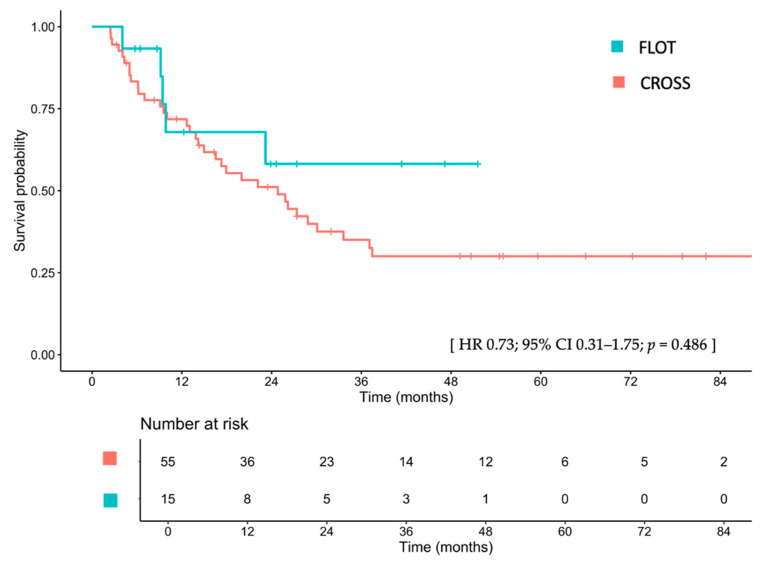
Disease-free survival between patients treated with FLOT and those treated with CROSS. FLOT, fluorouracil, leucovorin, oxaliplatin, and docetaxel; CROSS, chemoradiotherapy for esophageal cancer followed by surgery study; CI, confidence interval; HR, hazard ratio.

The median EFS was not reached (IQR 9.8–NA) in patients treated with FLOT, compared to 24.8 months (IQR 14.9–37.1) in those treated with CROSS (HR 0.73; 95% CI, 0.31–1.75; *p* = 0.486; Figure 2). After adjusting for sex, age, ECOG PS, clinical T stage, clinical N stage, tumor differentiation, tumor location, and receipt of surgical resection, the adjusted HR for EFS was 1.26 (95% CI, 0.33–4.82; *p* = 0.737). Detailed univariate and multivariable Cox proportional hazard analyses are provided in Table S1. The 1-, 2-, and 3-year EFS rates for the FLOT group were 63.0%, 54.0%, and 54.0%, respectively, compared with 71.8%, 51.1%, and 35.0% for the CROSS group. Among patients treated with CROSS who had residual disease (non-pCR), those who received adjuvant nivolumab had a median EFS that was 30.1 versus 26.2 months for those who did not receive nivolumab (HR 0.83; 95% CI, 0.19–3.64; *p* = 0.804; Figure S4).

### 3.4. Response Rates

Regarding the response rates (Table 3), the ORR was 60.0% in the FLOT group and 81.8% in the CROSS group, respectively (*p* = 0.436). Two patients (3.6%) in the CROSS group experienced disease progression following the completion of preoperative CCRT prior to surgery. Patients who achieved a complete response had a median OS that was not reached, whereas those with partial response, stable disease, and progressive disease had a median OS of 38.4, 30.3, and 9.9 months, respectively (Figure S5).

### 3.5. Pathological Response Rates

Pathological responses following surgery are summarized in Table 4. Among patients treated with FLOT, pathological T and N downstaging from the initial clinical stage were observed in 38.5% and 0% of patients, respectively, compared to 69.4% and 35.3% in those treated with CROSS. A pCR was achieved in 0% of the FLOT-treated patients and in 13.9% of those who received CROSS. Furthermore, patients in the CROSS group demonstrated a higher proportion of favorable pathological tumor regression grades (TRG 0–2) compared to those in the FLOT group.

### 3.6. Pattern of Recurrence and Subsequent Treatment

Recurrent disease was observed in 4 patients (26.7%) in the FLOT group and 27 patients (49.1%) in the CROSS group. Local recurrence was more frequent in patients treated with the FLOT regimen than in those treated with the CROSS protocol (50.0% vs. 22.2%, Table S2). Among the patients with recurrent disease, 75.0% and 55.5% in the FLOT and CROSS groups, respectively, received palliative systemic chemotherapy. The details of the treatment regimens are provided in Table S2.

### 3.7. Postoperative Complications

Among the patients who underwent radical surgery, those treated with FLOT experienced a higher incidence of surgical complications than those treated with CROSS, whereas non-surgical complications were more common in the CROSS group (Table S3). One patient in each group died of postoperative complications within 30 days of surgery.

### 3.8. Systemic Treatment Toxicities

A summary of the treatment-related toxicities is presented in Table 5. Although there was no significant difference in the overall hematologic toxicities, patients treated with CROSS experienced higher rates of neutropenia and leukopenia than those treated with FLOT. However, the incidence of febrile neutropenia was similar between the groups. Notably, all patients in the FLOT group received primary prophylaxis with G-CSF. Non-hematological toxicities were significantly more frequent in the FLOT group than in the CROSS group. Adverse events were predominantly mild, grades 1–2.

## 4. Discussion

This study presents a real-world comparative analysis of two established multimodal treatment strategies, perioperative chemotherapy with the FLOT regimen and preoperative chemoradiotherapy according to the CROSS protocol, in patients with localized esophageal and EGJ adenocarcinomas. Our findings indicated that both approaches resulted in comparable OS and EFS, supporting their continued use as viable treatment options in clinical practice. Notably, the CROSS protocol was associated with a higher ORR, more pronounced tumor downstaging, and a higher pCR rate, while demonstrating a more favorable toxicity profile than the FLOT.

Previous studies investigating the role of adding CCRT to pre- or perioperative chemotherapy have not demonstrated a significant OS benefit [23,25,30]. The NeoRes-I trial, which compared preoperative chemotherapy with cisplatin/5FU (CF) to CCRT with CF in resectable esophageal and EGJ cancers, reported no significant difference in OS between the two groups [23]. Similarly, the POET trial, which compared preoperative CF with preoperative CF followed by CCRT using cisplatin and etoposide, and the TOPGEAR trial, which compared perioperative epirubicin/cisplatin/5FU (ECF) or FLOT with or without preoperative CCRT using 5-FU, failed to show significant improvements in OS [25,30]. Notably, the TOPGEAR trial primarily included patients with gastric cancer, and only 27% had EGJ cancers, including Siewert types II and III. Nonetheless, higher pCR rates and improved local progression-free survival have consistently been observed in patients receiving concurrent radiotherapy. Hence, FLOT and CROSS remain the two main established standard treatment strategies for localized esophageal and EGJ adenocarcinoma [8,19,22]. Perioperative FLOT has improved OS compared with the ECF regimen [14], whereas preoperative CCRT according to the CROSS protocol has demonstrated improved OS compared with surgery alone [13].

To date, only two randomized controlled trials (RCTs) have directly compared the FLOT and CROSS; however, the results have been conflicting. The Neo-AEGIS trial, although limited by the fact that only 15% of patients received FLOT, reported comparable OS and DFS between the two arms but a higher pCR rate in the CROSS arm [24]. In contrast, the ESOPEC trial favored FLOT in terms of OS, DFS, and pCR [26]. However, it is important to note that the treatment intensity in the CROSS arm of the ESOPEC trial was lower than that in the original CROSS trial [13], with more frequent dose reductions and treatment discontinuations. Additionally, tumors in the ESOPEC trial were likely more extensive diseases, as the original CROSS trial excluded patients with cT4 disease and included a greater proportion of cT1–T2 tumors but fewer cN+ cases compared to ESOPEC. Notably, neither Neo-AEGIS nor ESOPEC incorporated adjuvant nivolumab for patients without pCR, which may have disadvantaged the CROSS arm under the current standard-of-care practices [27]. Our real-world findings are consistent with those of Neo-AEGIS in demonstrating no significant difference in OS or EFS between the two regimens, while also showing higher pCR rates and greater tumor downstaging with CROSS.

The similar OS and EFS observed between the FLOT and CROSS groups in our study, in contrast to the findings from ESOPEC, may be attributable to differences in sample size, follow-up duration, surgical resection rate, pCR rate, baseline ECOG PS, treatment completion rate, and the use of adjuvant nivolumab. The median OS in our FLOT group (30.3 months) was lower than that reported in the pivotal FLOT4-AIO trial (50.0 months) and the ESOPEC trial (66.0 months) [14,26]. Additionally, the 1-, 2-, and 3-year OS rates in our cohort (78.8%, 61.3%, and 46.0%, respectively) were slightly lower than those reported in FLOT4-AIO (84%, 68%, and 57%, respectively) and ESOPEC (82%, 65%, and 57.4%, respectively). A relatively small sample size and a shorter median follow-up time in the FLOT arm (13.4 months) in our study may have contributed to the lower observed OS, as many patients in our cohort had not yet reached the time point for key survival events. Notably, the median follow-up time in the FLOT group was shorter than the estimated median OS. This likely reflects the immature survival data, as the upper bound of the CI for OS was not reached and the number of events was limited at the time of analysis. In contrast, the median follow-up duration in the FLOT4-AIO and ESOPEC trials was 43 and 55 months, respectively. Furthermore, the lower surgical resection rate (86.7%) and absence of pCR (0%) in our FLOT group compared to 97% and 15.6% in FLOT4-AIO and 99.5% and 16.7% in ESOPEC may have further contributed to the limited survival outcomes.

In the CROSS cohort, the median OS was 37.5 months, which is slightly shorter than the 49.4 months reported in the original CROSS trial [13] and the 49.2 months in Neo-AEGIS [24], but comparable to the 37.0 months reported in ESOPEC [26]. This difference may be attributed to the lower rate of curative-intent surgery in our cohort (67.3%) than in the CROSS (94%), Neo-AEGIS (93.8%), and ESOPEC (98.9%) trials, as well as the lower proportion of patients with an ECOG PS of 0 (60% vs. 81% in CROSS, 83% in Neo-AEGIS, and 71.9% in ESOPEC). Importantly, patients who underwent surgery after CROSS showed a trend toward improved OS compared with those who did not (44.1 vs. 19.6 months), reaffirming the critical role of surgery in achieving durable outcomes in this complex disease setting. Although organ preservation is currently debated, with CCRT emerging as a potentially more suitable strategy than perioperative chemotherapy, strong supporting evidence is lacking. Therefore, decisions to omit surgery are primarily based on clinical discretion or multidisciplinary team discussions [31]. In our study, the pCR rate was 13.5%, which is comparable to the rates in the ESOPEC (10.1%) and Neo-AEGIS (12%) trials but lower than that in the original CROSS trial (23% in the adenocarcinoma subgroup). Treatment completion may have influenced this outcome, as 92.7% of patients in our cohort completed all five cycles of chemoradiotherapy, compared to 67.7% in the ESOPEC, 87% in the Neo-AEGIS, and 91% in the original CROSS trials.

A possible explanation for the relatively lower OS observed in the CROSS arm of our study is the lower surgical resection rate. In contrast, the comparable OS in ESOPEC, despite the higher surgery rate, may be partly explained by the lower treatment completion rate and the absence of adjuvant nivolumab. In our cohort, 19% of the eligible patients received adjuvant nivolumab, whereas the remainder did not, mostly because data collection predated its widespread adoption. A trend toward improved survival was observed among patients with non-pCR who received adjuvant nivolumab, suggesting that this agent may play a key role in prolonging survival in the CROSS group. Additionally, 20% of the patients in our CROSS group were HER2-positive, a subgroup associated with more aggressive tumor biology and poorer prognosis [32]. In contrast, none of the patients in the FLOT group were HER2-positive, which may have contributed to the survival differences between the groups. However, HER2 status has not been reported in previous major trials [13,14,26], and targeted therapy in this setting remains under investigation. Finally, post-recurrence treatment is another factor that may influence OS. A higher proportion of patients in the FLOT group received palliative systemic therapy upon progression (75.0% vs. 55.5% in the CROSS group), which may have contributed to the survival differences and is a variable rarely reported in previous trials [13,14,26].

Since OS and EFS were similar between the two treatment groups, factors such as postoperative mortality and morbidity, toxicity profiles, and patient tolerance were important in guiding treatment choices. In our study, the FLOT group showed a higher rate of surgical complications, whereas non-surgical complications were more common among patients treated with CROSS. The CROSS regimen was associated with higher incidences of leukopenia and neutropenia than the FLOT regimen. Nonetheless, these hematological toxicities were predominantly grades 1–2 and did not significantly affect treatment delivery, as reflected by the high treatment completion rate of 92.7% in the CROSS cohort. However, non-hematological toxicities, particularly nausea, mucositis, peripheral neuropathy, and hand–foot syndrome, were more frequently observed in the FLOT group and often contributed to treatment intolerance. Consequently, only 46.7% of the patients receiving FLOT were able to complete all planned treatment cycles. These findings align with prior reports and underscore the real-world challenges of delivering full-course FLOT outside clinical trial settings [14,26,33].

Molecular characterization is increasingly recognized as relevant to treatment selection for esophageal and EGJ adenocarcinoma. Biomarkers such as HER2 amplification, PD-L1 expression, and mismatch repair deficiency inform prognosis and therapeutic options, particularly in advanced disease [34]. In the localized setting, evidence from esophageal squamous cell carcinoma suggests that molecular classifiers can add prognostic value and refine risk stratification [35,36]. In our cohort, biomarker testing was incomplete and non-uniform, and the sample size was limited, precluding robust molecular analyses. Future adenocarcinoma-focused studies should integrate comprehensive molecular profiling to evaluate prognostic and predictive roles within multimodal strategies and to further personalize perioperative treatment.

A key strength of this study is that it is the first real-world analysis to directly compare perioperative chemotherapy with FLOT versus preoperative CCRT following the CROSS protocol in patients with localized esophageal and EGJ adenocarcinoma. Our real-world data offer valuable insights into the effectiveness and tolerability of both regimens outside the controlled environment of clinical trials. Moreover, our study uniquely included patients treated with adjuvant nivolumab in the CROSS arm, a factor not incorporated in previous studies. Despite the retrospective design, we employed multivariate Cox proportional hazards models to adjust for potential confounding factors. However, this study has some limitations. The single-center retrospective design inherently introduces a modest sample size, risks of selection bias, and incomplete data collection. Additionally, the relatively short follow-up period in the FLOT cohort may have led to an underestimation of long-term outcomes such as median survival. Given the modest sample size, particularly in the FLOT cohort, and the limited follow-up, these findings should be interpreted with caution; the study may be underpowered for definitive conclusions. A multicenter study with a substantially larger FLOT cohort and longer follow-up would increase the precision of effect estimates. This could uncover modest between-strategy differences or interactions, whereas persistent overlap of survival curves would support comparable effectiveness. Inclusion of multiple hospitals would also enhance generalizability and enable formal assessment of center-level heterogeneity using mixed-effects and propensity-based approaches. These extensions were beyond the scope of the present single-center cohort but represent a logical next step for future work.

## 5. Conclusions

This study reinforces the idea that preoperative CCRT with the CROSS regimen remains an effective treatment option for patients with localized esophageal and EGJ adenocarcinomas, showing no significant differences in OS or EFS compared with perioperative FLOT. This evidence may guide clinicians toward developing more patient-specific treatment strategies in routine practice. However, CROSS demonstrated higher pCR rates, greater tumor downstaging, and better treatment tolerability with fewer non-hematologic toxicities, suggesting it may be particularly suitable for patients with bulky tumors or limited PS (ECOG ≥ 1). Treatment selection is complex and should be guided by multidisciplinary discussions, patient preferences, tumor characteristics, and the availability of adjuvant immunotherapy. Further prospective, multicenter studies with larger cohorts, longer follow-up durations, and the incorporation of modern immunotherapy strategies are warranted to better define the optimal multimodal treatment approach for this patient population.

## Figures and Tables

**Table 1:** Baseline characteristics of the patients.

Characteristics	FLOT (*n* = 15)	CROSS (*n* = 55)	*p*-Value
Age, years (SD)	62.7 (9.7)	66.8 (9.4)	0.141
Age ≥ 65 years, *n* (%)	7 (46.7)	36 (65.5)	0.305
Male, *n* (%)	12 (80.0)	49 (89.1)	0.392
BMI, *n* (%)			0.809
18.5–24.9 kg/m^2^	6 (40.0)	18 (32.7)	
25.0–29.9 kg/m^2^	6 (40.0)	22 (40.0)	
≥30 kg/m^2^	3 (20.0)	15 (27.3)	
ECOG PS, *n* (%)			0.135
0	12 (80.0)	28 (50.9)	
1	3 (20.0)	26 (47.2)	
2	0 (0)	1 (1.8)	
Comorbidities, *n* (%) #	7 (46.7)	41 (74.5)	0.059
Hypertension	5 (33.3)	34 (61.8)	
Diabetes mellitus	3 (20.0)	6 (10.9)	
Cirrhosis	1 (6.7)	0 (0)	
Chronic kidney disease	0 (0)	3 (5.5)	
COPD	0 (0)	6 (10.9)	
Coronary artery disease	0 (0)	9 (16.4)	
Cerebrovascular disease	0 (0)	1 (1.8)	
Others	1 (6.7)	11 (20.0)	
Clinical T stage, *n* (%)			0.012
T2	3 (20.0)	4 (7.3)	
T3	10 (66.7)	51 (92.7)	
T4	2 (13.3)	0 (0)	
Clinical N stage, *n* (%)			0.427
N0	10 (66.7)	28 (50.9)	
N+	5 (33.3)	27 (49.1)	
Tumor location, *n* (%)			<0.001
Distal esophagus	1 (6.7)	26 (47.3)	
EGJ, Siewert I	0 (0)	14 (25.5)	
EGJ, Siewert II	14 (93.3)	15 (27.3)	
Tumor differentiation, *n* (%)			0.681
Well	0 (0)	3 (5.5)	
Moderately	5 (33.3)	23 (41.8)	
Poorly	10 (66.7)	29 (52.7)	
HER2 overexpression status, *n* (%)	*n* = 15	*n* = 40	0.091
Positive	0 (0)	8 (20.0)	
Negative	15 (100)	32 (80.0)	
MMR status, *n* (%)	*n* = 15	*n* = 38	0.568
Proficient	15 (100)	34 (89.5)	
Deficient	0 (0)	4 (10.5)	
PD-L1 status, *n* (%)			0.355
CPS ≥1	3 (20.0)	5 (9.1)	
Not tested	12 (80.0)	50 (90.9)	
Preoperative feeding tube, *n* (%)			0.005
Percutaneous jejunostomy	2 (13.3)	29 (52.7)	
Nasojejunal tube	1 (6.7)	1 (1.8)	
Nasogastric tube	1 (6.7)	0 (0)	
Baseline laboratory values			
Hemoglobin, g/dL (SD)	12.7 (2.0)	13.5 (1.8)	0.147
Albumin, g/dL (IQR)	3.9 (3.8, 4.2)	3.8 (3.5, 4.0)	0.175
CrCl, mL/min (SD)	96.5 (23)	103.5 (29)	0.517

# There could be more than one answer. SD, standard deviation; BMI, body mass index; ECOG, Eastern Cooperative Oncology Group; PS, performance status; COPD, chronic obstructive pulmonary disease; EGJ, esophagogastric junction; MMR, mismatch repair; IQR, interquartile range; CrCl, creatinine clearance; FLOT, fluorouracil, leucovorin, oxaliplatin, and docetaxel; CROSS, chemoradiotherapy for esophageal cancer followed by surgery study.

**Table 2:** Treatment information.

	FLOT (*n* = 15)	CROSS (*n* = 55)
Number of preoperative cycles, *n* (%)		
Complete	14 (93.3)	51 (92.7)
Incomplete	1 (7.7)	4 (7.3)
Number of postoperative cycles, *n* (%)		- - -
Complete	7 (46.7)	
Incomplete	1 (7.7)	
No	7 (46.7)	
Dose reduction, *n* (%)	9 (60.0)	1 (1.8)
Discontinuation, *n* (%) *		
Complete	7 (46.7)	52 (94.5)
Postoperative disease progression	1 (6.7)	0 (0)
Death	2 (13.3)	0 (0)
Declined ECOG PS	2 (13.3)	1 (1.8)
Patient’s preference	2 (13.3)	0 (0)
Infection	0 (0)	1 (1.8)
Prolonged neutropenia	0 (0)	1 (1.8)
Postoperative complication	1 (6.7)	0 (0)
Median time to surgery after preop treatment, months (range)	2.07 (1.60–4.30)	2.23 (0.92–5.32)
Resection, *n* (%)	13 (86.7)	37 (67.3)
Resection status, *n* (%)		
R0	13 (100)	36 (97.3)
R1	0 (0)	1 (2.7)
Resection type, *n* (%)		
Ivor Lewis esophagectomy	2 (15.4)	36 (97.3)
Esophagectomy and gastrectomy	3 (23.1)	0 (0)
Total gastrectomy	5 (38.5)	1 (2.7)
Partial gastrectomy	3 (23.1)	0 (0)
Cause of non-resection, *n* (%)		
Progressive disease (metastasis)	0 (0)	5 (9.1)
Death	1 (6.7)	0 (0)
Patient’s denial	0 (0)	1 (1.8)
Declined ECOG PS	0 (0)	8 (14.5)
Unresectable	1 (6.7)	4 (7.3)
Adjuvant nivolumab	-	6 (10.9)

* Significant difference between FLOT and CROSS (*p* < 0.05). ECOG, Eastern Cooperative Oncology Group; PS, performance status; FLOT, fluorouracil, leucovorin, oxaliplatin, and docetaxel; CROSS, chemoradiotherapy for esophageal cancer followed by surgery study.

**Table 3:** Response rates.

Response	FLOT (*n* = 15)	CROSS (*n* = 55)
Objective response rate	9 (60.0)	45 (81.8)
Complete response	1 (6.7)	1 (1.8)
Partial response	8 (53.3)	44 (80.0)
Stable disease	6 (40.0)	8 (14.6)
Progressive disease	0 (0)	2 (3.6)

FLOT, fluorouracil, leucovorin, oxaliplatin, and docetaxel; CROSS, chemoradiotherapy for esophageal cancer followed by surgery study.

**Table 4:** Pathological response rates.

	FLOT (*n* = 13)	CROSS (*n* = 37)
Pathological T stage, *n* (%)		
ypT0	0 (0)	5 (13.5)
ypT1	4 (30.8)	13 (35.1)
ypT2	2 (15.3)	9 (24.3)
ypT3	4 (30.8)	9 (24.3)
ypT4	3 (23.1)	1 (2.7)
Downstaging T stage at least 1 or more stages, *n* (%)	5 (38.5)	26 (70.3)
Pathological N stage, *n* (%)		
ypN0	6 (46.1)	22 (59.5)
ypN1	3 (23.1)	8 (21.6)
ypN2	1 (7.7)	6 (16.2)
ypN3	3 (23.1)	1 (2.7)
Downstaging N stage from cN+ to ypN0, *n* (%)	0 (0)	7/17 (41.2) #
Pathological complete response, *n* (%)	0 (0)	5 (13.5)
Pathological tumor regression grade, *n* (%) *,**		
Grade 0	0 (0)	5 (13.5)
Grade 1	0 (0)	10 (27.0)
Grade 2	6 (46.2)	20 (54.1)
Grade 3	7 (53.8)	2 (5.4)

# Seven of the 17 patients with initial cN+ underwent resection. * Significant difference between the FLOT and CROSS groups (*p* < 0.05). ** Pathological tumor regression grade was evaluated by the modified Ryan system [29]. FLOT, fluorouracil, leucovorin, oxaliplatin, and docetaxel; CROSS, chemoradiotherapy for esophageal cancer followed by surgery study.

**Table 5:** Systemic treatment toxicities.

Toxicities	Perioperative Chemotherapy (*n* = 15)		Preoperative CCRT (*n* = 49)	
	All Grades, *n* (%)	Grades 3–4, *n* (%)	All Grade, *n* (%)	Grades 3–4, *n* (%)
Hematologic				
Anemia	3 (20.0)	0 (0)	4 (8.2)	0 (0)
Thrombocytopenia	0 (0)	0 (0)	3 (6.1)	0 (0)
Leukopenia	1 (6.7)	1 (6.7)	12 (24.5)	4 (8.2)
Neutropenia	1 (6.7)	1 (6.7)	12 (24.5)	4 (8.2)
Febrile neutropenia	1 (6.7)	1 (6.7)	2 (4.1)	2 (4.1)
Non-hematologic				
Nausea	11 (73.3) *	0 (0)	19 (38.8)	0 (0)
Vomiting	3 (20.0)	0 (0)	5 (10.2)	0 (0)
Mucositis	3 (20.0) *	0 (0)	1 (2.0)	0 (0)
Diarrhea	9 (60.0) *	0 (0)	3 (6.1)	0 (0)
Fatigue	15 (100) *	0 (0)	30 (61.2)	1 (2.0)
Peripheral neuropathy	10 (66.7) *	0 (0)	3 (6.1)	0 (0)
Infection	1 (6.7)	0 (0)	12 (24.5)	0 (0)
Hand–foot syndrome	3 (20.0) *	0 (0)	0 (0)	0 (0)
Pneumonitis	0 (0)	0 (0)	0 (0)	0 (0)

* Significant difference between the FLOT and CROSS groups (*p* < 0.05). FLOT, fluorouracil, leucovorin, oxaliplatin, and docetaxel; CROSS, chemoradiotherapy for esophageal cancer followed by surgery study; CCRT, concurrent chemoradiotherapy.

## Data Availability

The datasets generated and/or analyzed during the current study are not publicly available due to institutional data protection policies but are available from the corresponding author on reasonable request and with appropriate institutional approval.

## Supplementary Materials

The following supporting information can be downloaded at: https://www.mdpi.com/article/10.3390/cancers17182962/s1, Figure S1: Flow diagram of patient selection; Figure S2: Overall survival between patients who received adjuvant nivolumab and those who did not; Figure S3: Overall survival between patients who underwent resection and those who did not in the CROSS group; Figure S4: Disease-free survival between patients who received adjuvant nivolumab and those who did not; Figure S5: Overall survival by response; Table S1: Univariate and multivariable Cox proportional hazards analyses for OS and EFS; Table S2: Pattern of recurrence and subsequent treatment; Table S3: Postoperative complications.

## Abbreviations

The following abbreviations are used in this manuscript:

BMI	Body mass index
CCRT	Concurrent chemoradiotherapy
CI	Confidence interval
CROSS	Chemoradiotherapy for esophageal cancer followed by surgery study CT
CT	Computed tomography
EFS	Event-free survival
DFS	Disease-free survival
ECOG	Eastern Cooperative Oncology Group; EGJ, esophagogastric junction
FLOT	Fluorouracil, leucovorin, oxaliplatin, and docetaxel
G-CSF	Granulocyte colony-stimulating factor
HR	Hazard ratio
IQR	Interquartile ranges
ORR	Objective response rate
OS	Overall survival
pCR	Pathological complete response
PET	Positron emission tomography
RCTs	Randomized controlled trials
SD	Standard deviations
