# Supplementary material for: Real-World Outcomes Between Perioperative Chemotherapy (FLOT) and Preoperative Concurrent Chemoradiotherapy (CROSS) in Localized Esophageal and Esophagogastric Junction Adenocarcinoma: A Retrospective Cohort Study
Source: Cancers (Basel). 2025 Sep 10;17(18):2962. doi: 10.3390/cancers17182962 (PMC12468997; doi:10.3390/cancers17182962)
Supplement: Supplementary file 1 [file cancers-17-02962-s001.zip › cancers-3836855-supplementary.pdf]

Supplementary data

Figure S1 Flow diagram of patient selection

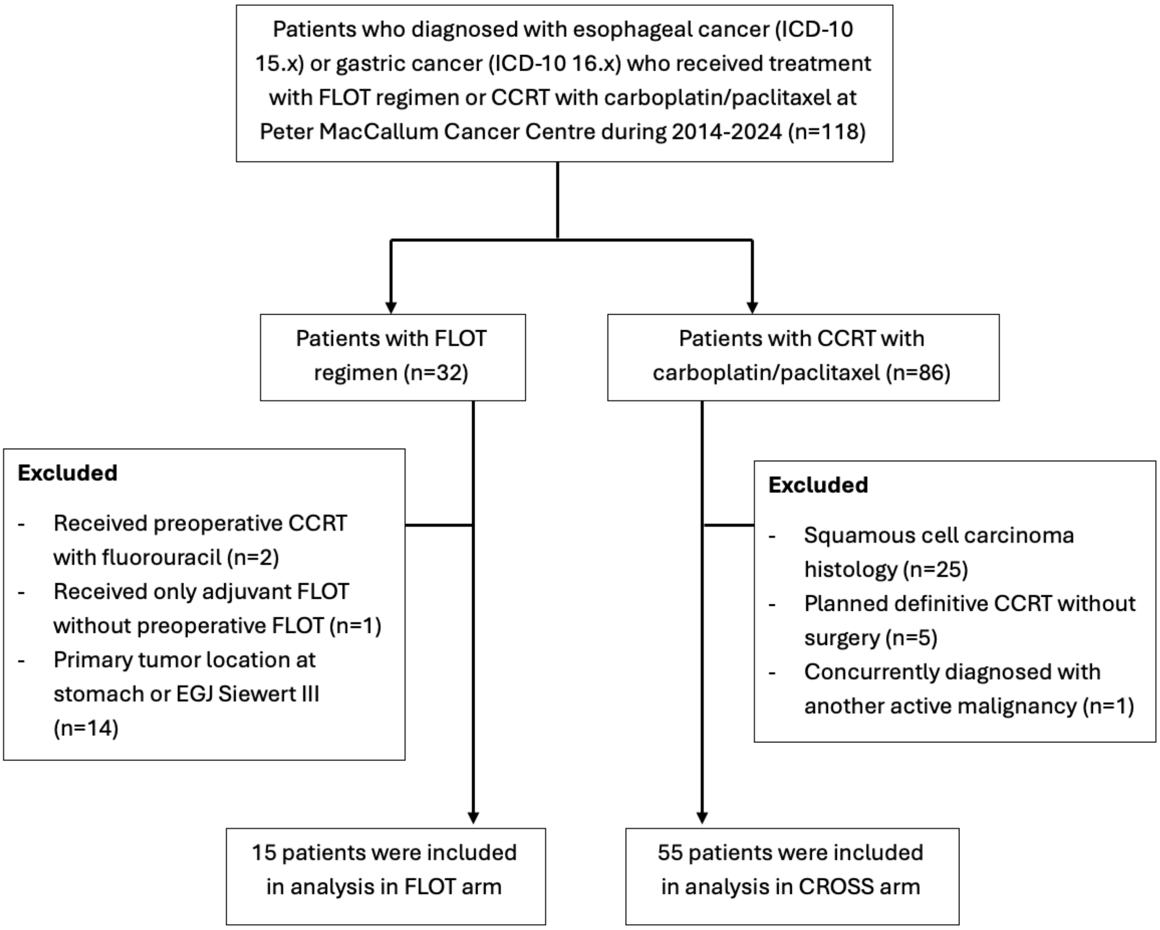

FLOT, fluorouracil, leucovorin, oxaliplatin, and docetaxel; CCRT, concurrent chemoradiotherapy; EGJ, esophagogastric junction; CROSS, chemoradiotherapy for esophageal cancer followed by surgery study.

Figure S2: Overall survival between patients who received adjuvant nivolumab and those who did not

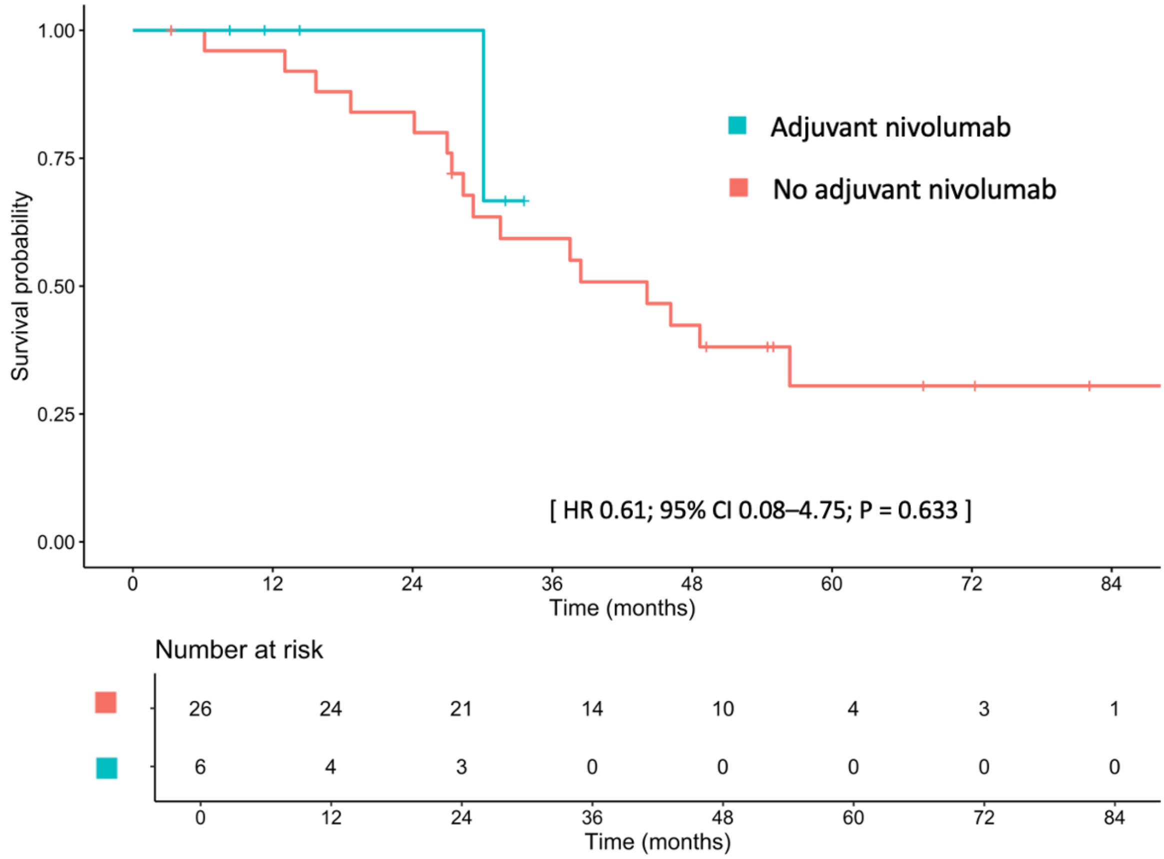

CI, Confidence interval; HR, Hazard ratio

Figure S3 Overall survival between patients who underwent resection and those who did not

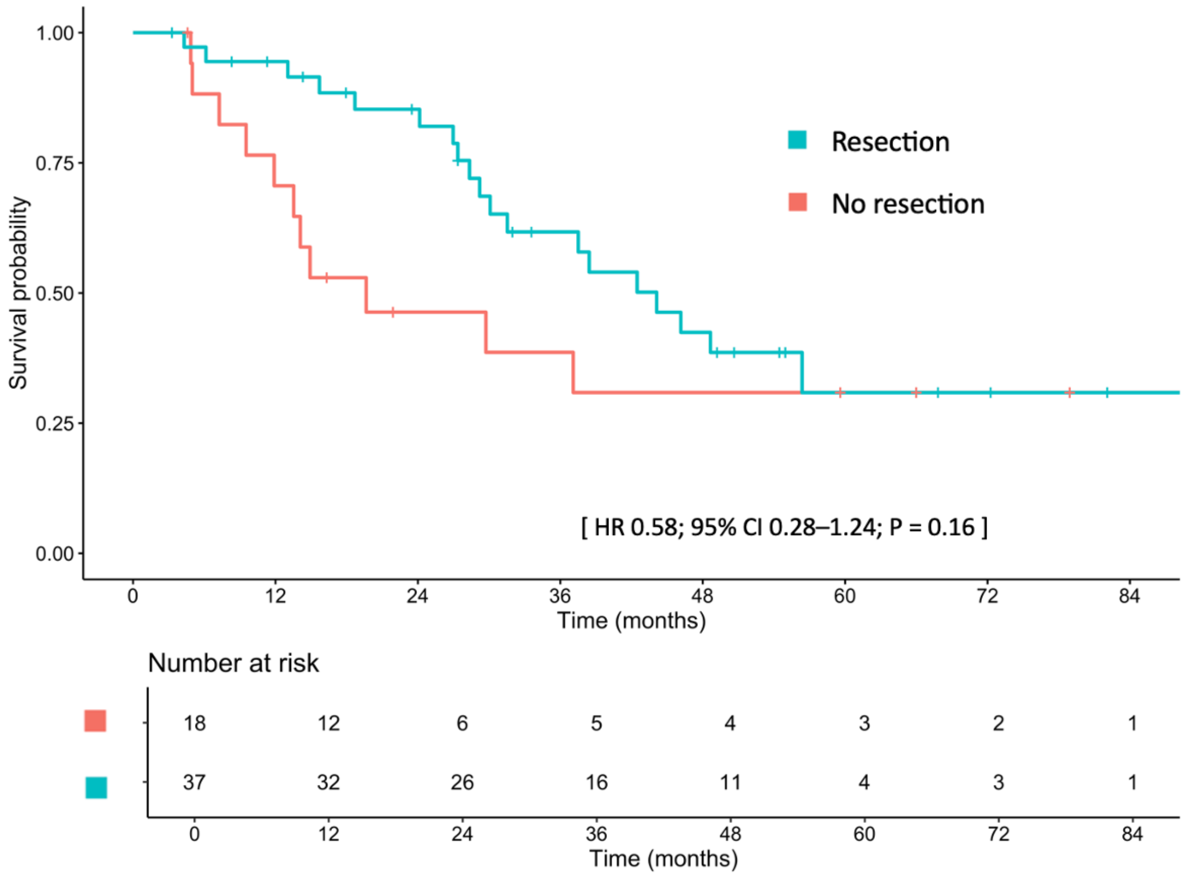

CI, Confidence interval; HR, Hazard ratio

Figure S4 Disease-free survival between patients received adjuvant nivolumab and those who did not

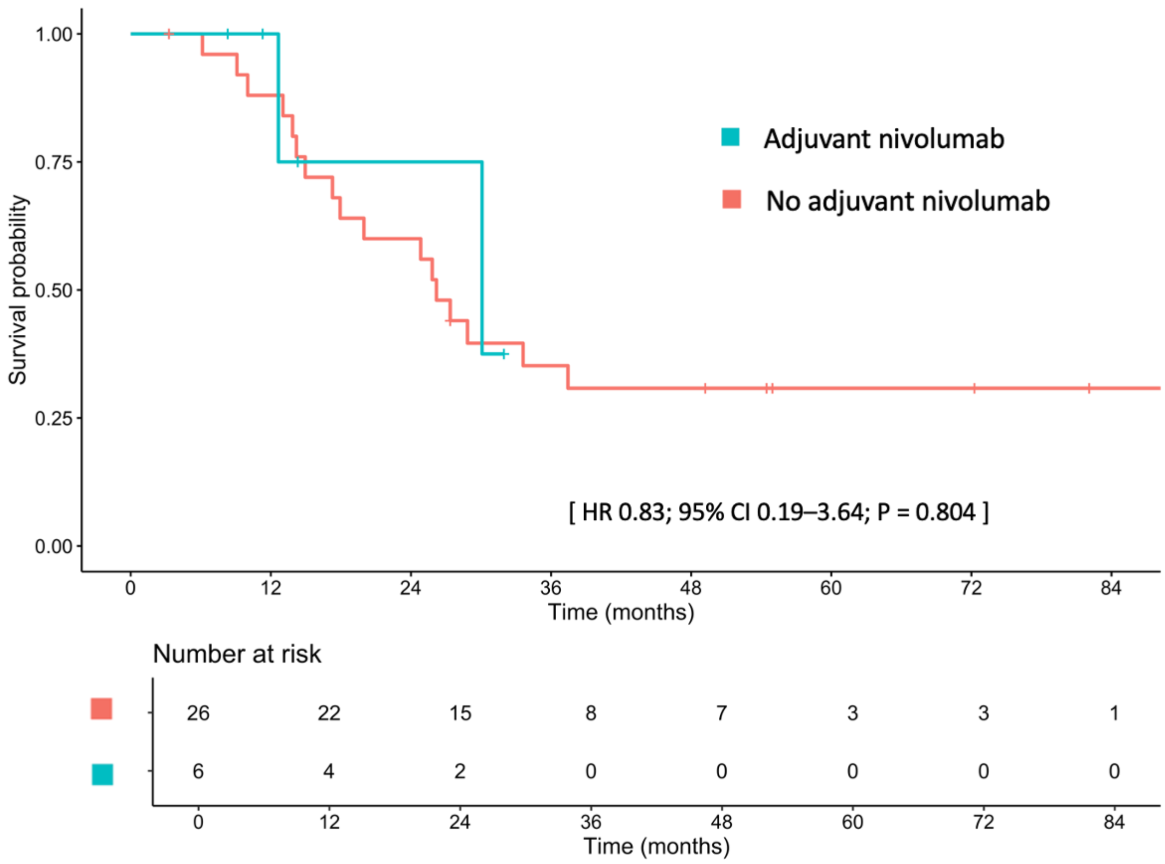

CI, Confidence interval; HR, Hazard ratio

Figure S5 Overall survival by response

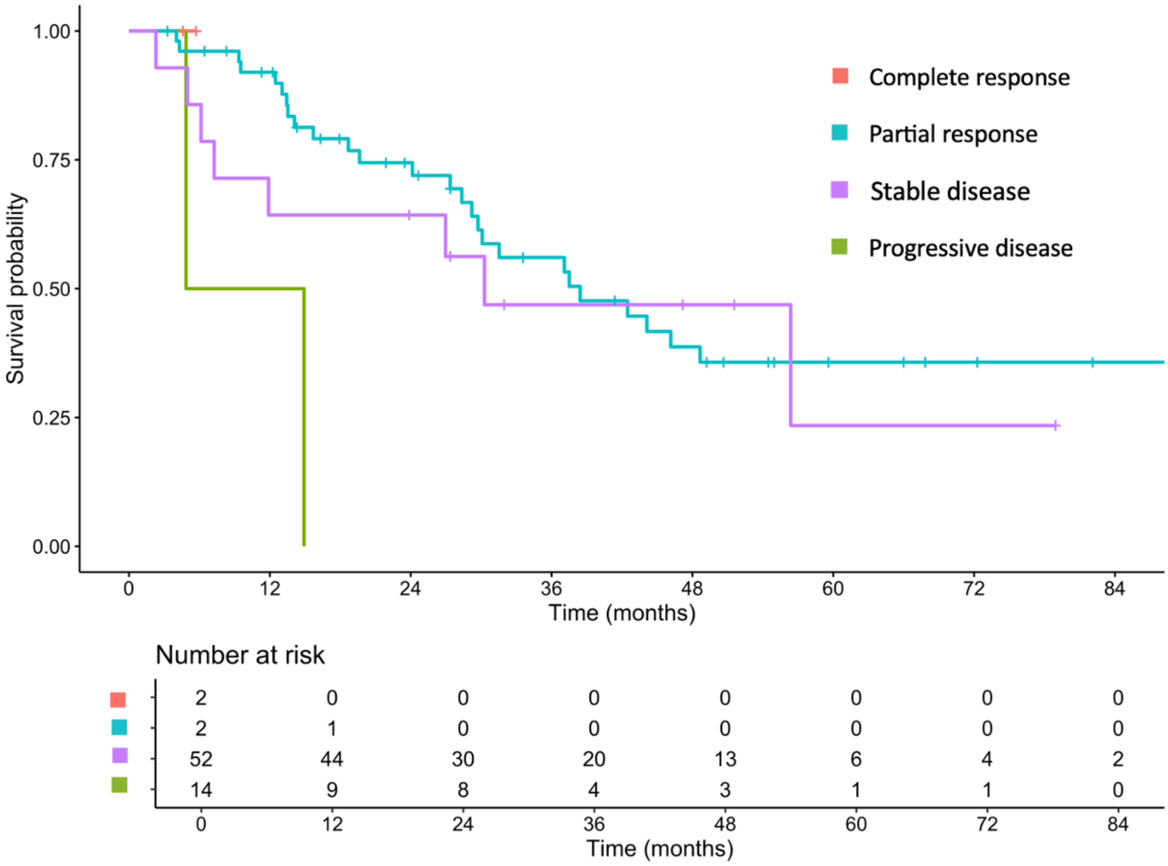

Table S1 Univariate and multivariable Cox proportional hazards analyses for OS and EFS.

Factors	OS		EFS	
	Univariate analysis, HR (95% CI)	Multivariate analysis, HR (95% CI)	Univariate analysis, HR (95% CI)	Multivariate analysis, HR (95% CI)
FLOT vs. CROSS	1.15 (0.48,2.79)	2.9 (0.66,12.84)	0.73 (0.31,1.75)	1.26 (0.33,4.82)
Male vs. Female	6.96 (0.96,50.88)	8.48 (0.91,78.65)	8.29 (1.14,60.39)	7.46 (0.85,65.62)
ECOG PS				
0	Ref	Ref	Ref	Ref
1	2.04 (1.04,3.99)	1.76 (0.67,4.63)	1.65 (0.88,3.09)	1.05 (0.41,2.7)
2	2.07 (0.27,15.8)	4.49 (0.33,60.73)	1.22 (0.16,9.15)	1.4 (0.11,17.09)
Age	0.99 (0.96,1.03)	0.97 (0.92,1.01)	0.99 (0.96,1.02)	0.96 (0.92,1.01)
cT stage				
cT2	Ref	Ref	Ref	Ref
cT3	2.25 (0.54,9.41)	1.68 (0.35,8.03)	2.64 (0.63,10.99)	1.63 (0.34,7.72)
cT4	7.1 (0.98,51.44)	1.87 (0.16,21.71)	6.89 (0.95,49.76)	2.77 (0.26,29.93)
cN1 vs cN0	1.47 (0.76,2.84)	1.45 (0.71,2.97)	1.76 (0.94,3.29)	1.57 (0.79,3.1)
Differentiation				
Well	Ref	Ref	Ref	Ref
Moderately	0.85 (0.19,3.79)	0.39 (0.05,2.76)	1.09 (0.25,4.77)	0.43 (0.07,2.72)
Poorly	1.16 (0.27,4.96)	0.53 (0.07,4.07)	1.52 (0.36,6.48)	0.55 (0.08,3.77)
Location				
Distal esophagus	Ref	Ref	Ref	Ref
EGJ Siewert I	0.99 (0.43,2.28)	1.25 (0.45,3.49)	0.76 (0.34,1.74)	0.95 (0.34,2.62)
EGJ Siewert II	0.69 (0.32,1.5)	0.69 (0.21,2.27)	0.58 (0.28,1.19)	0.77 (0.28,2.07)
Tumor resection	0.55 (0.28,1.09)	0.71 (0.23,2.21)	0.49 (0.26,0.95)	0.45 (0.15,1.34)

FLOT, fluorouracil, leucovorin, oxaliplatin, and docetaxel; CROSS, chemoradiotherapy for esophageal cancer followed by surgery study; OS, overall survival; EFS, event-free survival; ECOG, Eastern Cooperative Oncology Group; PS, performance status; cT, clinical T stage; cN, clinical N stage; EGJ, esophagogastric junction; HR, hazard ratio; CI, confidence interval; Ref, reference.

Table S2 Pattern of recurrence and subsequent treatment.

	Perioperative chemotherapy (n=4)	Preoperative CCRT (n=27)
Recurrence #		
Distant metastasis	4 (100.0)	26 (96.3)
Local recurrence	2 (50.0)	6 (22.2)
Palliative systemic therapy	3 (75.0)	15 (55.5)
FOLFOX	0 (0)	3 (11.1)
FOLFOX/nivolumab	2 (50.0)	6 (22.2)
FOLFOX/trastuzumab	0 (0)	3 (11.1)
FOLFOX/trastuzumab/nivolumab	0 (0)	1 (3.7)
Carboplatin/paclitaxel	0 (0)	1 (3.7)
EOX	0 (0)	1 (3.7)
Irinotecan	1 (25.0)	0 (0)
Unknown	0 (0)	4 (14.8)
No	1 (25.0)	8 (29.6)
Subsequent local treatment		
Palliative RT at metastatic site	0 (0)	5 (18.5)
Resection	0 (0)	1 (3.7)

could have more than one answer. RT, radiotherapy; CCRT, concurrent chemoradiotherapy

Table S3 Postoperative complications.

	Perioperative chemotherapy (n=13)	Preoperative CCRT (n=37)
Post op complication #	5 (38.5)	14 (37.8)
**Surgical**	4 (30.8)	6 (16.2)
Anastomotic leakage	3 (23.1)	3 (8.1)
Pelvic collection	1 (7.7)	0 (0)
Infected seroma	0 (0)	1 (2.7)
Gastric outlet obstruction	0 (0)	1 (2.7)
Tracheo-esophageal fistula	0 (0)	1 (2.7)
**Non-surgical**	3 (23.1)	10 (27.0)
Hospital acquired pneumonia	1 (7.7)	9 (24.3)
Acute pulmonary embolism	1 (7.7)	1 (2.7)
Atrial fibrillation	0 (0)	2 (5.4)
Sepsis	1 (7.7)	1 (2.7)
30-day post-operative mortality	1 (7.7)	1 (2.7)

#could have more than one complication in each patient. CCRT, concurrent chemoradiotherapy
